# Biochemical Toxicological Study of Insulin Overdose in Rats: A Forensic Perspective

**DOI:** 10.3390/toxics12010017

**Published:** 2023-12-23

**Authors:** Cunhao Bian, Xin He, Qi Wang, Zhe Zheng, Yongtai Zhang, Hongli Xiong, Yongguo Li, Mingzhu Zhao, Jianbo Li

**Affiliations:** 1 Department of Forensic Medicine, Faculty of Basic Medical Sciences, Chongqing Medical University, Chongqing 400016, China; 2022320004@stu.cqmu.edu.cn (C.B.); hx_isabella@163.com (X.H.); forensic.wangqi@cqmu.edu.cn (Q.W.); forensiczheng@sina.com (Z.Z.); zyongtai1536@163.com (Y.Z.); 15023159916@163.com (H.X.); 100399@cqmu.edu.cn (Y.L.); 102870@cqmu.edu.cn (M.Z.); 2 Chongqing Engineering Research Center of Criminal Investigation Technology, Chongqing 400016, China; 3 Chongqing Key Laboratory of Forensic Medicine, Chongqing 400016, China

**Keywords:** insulin overdose, forensic medicine, hypoglycemia, toxicology

## Abstract

Due to nonspecific pathological changes and the rapid degradation of insulin in postmortem blood samples, the identification of the cause of death during insulin overdose has always been a difficulty in forensic medicine. At present, there is a lack of studies on the toxicological changes and related mechanisms of an insulin overdose, and the specific molecular markers of insulin overdose are still unclear. In this study, an animal model of insulin overdose was established, and 24 SD rats were randomly divided into a control group, insulin overdose group, and a recovery group (*n* = 8). We detected the biochemical changes and analyzed the toxicological mechanism of an insulin overdose. The results showed that after insulin overdose, the rats developed irregular convulsions, Eclampsia, Opisthotonos, and other symptoms. The levels of glucose, glycogen, and C-peptide in the body decreased significantly, while the levels of lactate, insulin, and glucagon increased significantly. The decrease in plasma K^+^ was accompanied by the increase in skeletal muscle K^+^. The PI3K-AKT signaling pathway was significantly activated in skeletal muscle, and the translocation of GLUT4/Na^+^-K^+^-ATPase to sarcolemma was significantly increased. Rare glycogenic hepatopathy occurred in the recovery group after insulin overdose. Our study showed that insulin overdose also plays a role in skeletal muscle cells, mainly through the PI3K-Akt signaling pathway. Therefore, the detection of signaling pathway proteins of the skeletal muscle cell membrane GLUT4 and Na^+^-K^+^-ATPase has a certain auxiliary diagnostic value for forensic insulin overdose identification. Glycogen detection in the liver and skeletal muscle is important for the diagnosis of insulin overdose, but it still needs to be differentiated from other causes of death. Skeletal muscle has great potential for insulin detection, and the ratio of insulin to the C-peptide (I:C) can determine whether an exogenous insulin overdose is present.

## 1. Introduction

Insulin is an anabolic hormone produced by pancreatic β-cells and consists of two polypeptide chains, A and B, linked by disulfide bonds [1,2]. The main functions of insulin include regulating blood glucose levels and promoting glucose uptake by peripheral cells [1,2,3]. Insulin biosynthesis is performed by the cleavage and processing of the precursor single-chain molecule proinsulin to secrete equimolar masses of mature insulin and C-peptide into the blood [2,3,4]. The blood glucose level is the only major physiological stimulus of insulin secretion, and insulin and C-peptide secretion almost ceases when the blood glucose level stabilizes between 2.5 and 3.33 mmol/L [2,3].

Insulin is the most common drug used in the clinical treatment of diabetes [3], so insulin overdose is mostly an accidental treatment for diabetic patients, including the miscalculation of dosage and incorrect use of insulin specifications [1,5,6,7]. An insulin overdose can lead to hypoglycemia, with blood glucose levels below 3.9 mmol/L (70 mg/dL) defined as clinical hypoglycemia and blood glucose levels below 3 mmol/L (54 mg/dL) defined as clinically important hypoglycemia [7,8,9]. The clinical symptoms of hypoglycemia can be summarized into the following two aspects: sympathetic hyperactivity, including sweating, hunger, palpitation, and brain dysfunction, including confusion, drowsiness, impaired coordination, visual impairment, paresthesia, serious convulsions, coma, and even death [5,6,8,9,10]. The exact mechanism by which hypoglycemia leads to sudden death is unknown, but arrhythmias and cerebral epilepsy appear to play a major role [8,11,12,13].

Insulin overdose also appears in forensic cases, especially among medical personnel and families of diabetic patients, who can easily obtain insulin to commit suicide or murder [7,14,15,16,17]. In 1958, BIRKINSHAW reported the first insulin murder in history [18]. Since then, the number of insulin homicides has gradually increased, and some novelists have described insulin as “the perfect murder method” [17,19,20,21,22,23]. The identification of the cause of death after an insulin overdose has always been difficult in forensic medicine due to the lack of evidence left at the crime scene, the difficulty in finding insulin injection needle marks on the corpse’s surface, the lack of obvious pathological characteristics in autopsy, the rapid degradation of insulin caused by postmortem hemolysis, and the significant difference in postmortem blood biochemistry compared with antemortem conditions [14,15,17,20,21,23]. At present, the studies of insulin overdose are mostly limited to case reports and reviews of the literature [7,14,17,18,23], and there is a lack of research on the biochemical and toxicological mechanism of insulin overdose death, molecular markers related to insulin overdose, and alternative samples for insulin forensic determination.

The purpose of this experiment was to study the biochemical changes and toxicological mechanisms of insulin overdose through animal models and to search for specific molecular markers after insulin overdose so as to provide a reference for the clinical treatment of insulin overdose and theoretical support for the forensic diagnosis of insulin.

## 2. Materials and Methods

### 2.1. Experimental Animals

A total of 24 SPF male SD rats (8 weeks, 310 g ± 20 g) were purchased from Chongqing Medical University Laboratory Animal Center. All rats were maintained at 22–24 °C (12 h light/dark cycle) with sterile rat chow and water ad libitum. All rats were sacrificed or died under intraperitoneal sodic pentobarbital anesthesia (100 mg/kg, ip). All animal procedures were performed in accordance with animal care ethics, and all animal experiments for this study were approved by the Experimental Animal Care and Use Committee of Chongqing Medical University.

### 2.2. Experimental Design

**Figure 1:**
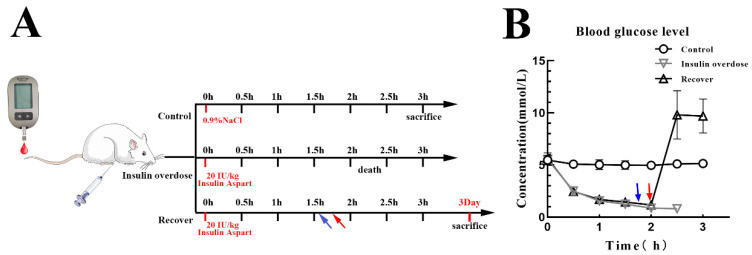
(**A**) Experimental protocol. Rats were divided into three groups: Control (*n* = 8), Insulin overdose (*n* = 8), Recovery (*n* = 8). Blood glucose was measured per 30 min in all rats. Control group rats were injected with normal saline (ip). Insulin overdose group rats were injected with insulin (20 IU/kg,ip). Recovery group rats were injected with insulin (20 IU/kg,ip) injected with a 0.3 mL mixture (50%-glucose: bicarbonate buffer = 1:1, ip) after blood glucose dropped to 1.5 mmol/L(27 mg/dL) (blue arrow), and intraperitoneally injected with 5 mL of 50%-glucose (red arrow). (**B**) Blood glucose in all rats was recorded within 3 h.

All rats fasted overnight before the experiment and were provided with pure water only. In total, 24 SD rats were randomly divided into the control group, insulin overdose group, and insulin overdose recovery group (*n* = 8). The rats in the insulin overdose group were intraperitoneally injected with 20 IU/kg of insulin (insulin aspart injection, Novonordisk A/S, Copenhagen, Denmark). Their blood glucose levels were measured every 30 min from the end of their tail veins using a glucometer (Accu-Chek-performa, Roche, Mannheim, Germany) until they died, and their physical signs were recorded throughout the experiment. The rats in the recovery group were intraperitoneally injected with 20 IU/kg of insulin; a 0.3 mL mixture of 50% glucose and bicarbonate buffer (1:1) was injected intraperitoneally when blood glucose dropped to 1.5 mmol/L (27 mg/dL); 5 mL of a 50% glucose solution (H50020071, TaiJi, Chongqing, China) was injected intraperitoneally for resuscitation if the rats developed convulsions or opisthotonos; they were fed in cages after their blood glucose levels rose above 3.9 mmol/L (70 mg/dL) and were sacrificed 3 days later. The rats in the control group were injected intraperitoneally with the same volume of sterile normal saline (BL158 A, Biosharp, Hefei, China) only; blood glucose levels were measured every 30 min, and the rats were sacrificed 3 h later. Plasma, the quadriceps femoris muscle, and the liver were collected from all rats and stored at −80 °C for further analysis. The experimental protocol is shown in Figure 1A.

### 2.3. Biochemical Index

The glucose content in liver and skeletal muscle tissue was determined using the glucose colorimetric method kit (E-BC-K234-M, Elabscience, Wuhan, China) according to the manufacturer’s instructions. Lactate levels in plasma and the skeletal muscle, potassium levels in plasma and the skeletal muscle, and ALT and AST levels in plasma were determined using kits (A019-2-1, C001-2-1, C009-2-1, C010-2-1, NanJing Jiancheng Bioengineering Institute, NanJing, China) according to the manufacturer’s instructions. The plasma total ketone body content was determined via ultraviolet spectrophotometry according to the manufacturer’s instructions (BC5060, Solarbio, Beijing, China). All tissue homogenates were obtained using a Cryogenic freezing grinder (JXFSTPRP-CL, JINXIN, Shanghai, China).

### 2.4. ELISA for Hormone Levels

Insulin in plasma and the skeletal muscle were determined using the Iso-Insulin ELISA kit (10-1128-01, Mercodia, Uppsala, Sweden) with standardized cross-reaction validation. Glucagon (MM-21295R1, MEIMIAN, Yancheng, China) and C-peptide (MM-0588R1, MEIMIAN, Yancheng, China) levels in the plasma were determined using reagent vendors standardized ELISA kits according to the manufacturer’s protocol.

### 2.5. Liver Pathology

Liver weights were determined by analytical balance. Liver tissues were fixed in Carnoy’s solution, embedded in paraffin, sectioned at 4 μm, and stained using H&E to observe pathological changes in the liver.

### 2.6. Glycogen Determination

The absolute glycogen content in fresh liver and skeletal muscle was determined using an anthrone method kit (A043-1-1, NanJing Jiancheng Bioengineering Institute, NanJing, China) according to the manufacturer’s instructions. Parts of the liver and skeletal muscle from the same site were fixed in Carnoy’s solution, embedded in paraffin, sectioned at 4 μm, and stained with PAS. Image J was used to analyze the degree of PAS staining.

### 2.7. Western Blotting Analysis

Skeletal muscle samples were ground and homogenized in a RIPA lysis buffer containing protease inhibitors and phosphatase inhibitors (P0013B, Beyotime, Shanghai, China), and total protein supernatants were obtained after centrifugation and protein concentrations were determined using the BCA protein assay kit (P0010, Beyotime, Shanghai, China). The protein supernatant was mixed with bromophenol blue and boiled at 95 °C for 10 min. Total proteins (20 ug) were separated via 10% SDS-PAGE gel electrophoresis and transferred onto PVDF membranes (PR05505, Immobilon, Darmstadt, Germany). After blocking with 5% free-fat milk for 2 h at room temperature, membranes were incubated with the primary antibodies against GSK3 (1:5000, ab185141, abcam, Cambridge, UK), Phosphorylated-GSK3-Ser9 (1:5000, ab75814, abcam, Cambridge, UK), AKT (1:1000, T55561F, Abmart, Shanghai, China), Phosphorylated-AKT-Ser473 (1:1000, T40067F, Abmart, Shanghai, China), Phosphorylated-AKT-Thr308 (1:1000, T40068F, Abmart, Shanghai, China), PI3K (1:5000, 60225-1-lg, proteintech, Wuhan, China), Phosphorylated-PI3K (1:1000, bs-3332R, Bioss, Beijing, China), GLUT4 (1:1000, 66846-1-lg, proteintech, Wuhan, China), Na^+^/K^+^-ATPase (1:5000, 14418-1-AP, proteintech, Wuhan, China), and β-actin (1:5000, 66009-1-lg, proteintech, Wuhan, China) overnight at 4 °C, then incubated with HRP secondary antibodies(1:2000, proteintech, Wuhan, China), detected using ECL(BL520B, Biosharp, Hefei, China), and visualized using the ChemiDoc imaging system(Bio-Rad, California, USA). Total protein normalization was performed using 10% acryl-amide TGX Stain-free^TM^ gels(Bio-Rad, California, USA). Protein levels were quantified using Image Lab 5.2.1 software. The mean density of protein bands was normalized to β-actin and control data, expressed as fold changes relative to the control.

### 2.8. Confocal Immunofluorescent Staining

Skeletal muscles were fixed in a 4% formaldehyde solution, embedded in paraffin, and sectioned at 4 μm for immunofluorescence staining to detect the expression and subcellular localization of GLUT4 and Na^+^/K^+^-ATPase. Sections were deparaffinized, antigen-repaired, blocked, and incubated with the anti-GLUT4 antibody (1:200, 66846-1-lg, proteintech, Wuhan, China) and anti-Na^+^/K^+^-ATPase antibody (1:400, 14418-1-AP, proteintech, Wuhan, China) overnight at 4 °C. After washing with PBS, sections were incubated with the fluorescent secondary antibody CoraLite594/CoraLite488 (1:100, proteintech, Wuhan, China) for 2 h at room temperature, washed again with PBS, stained with nuclei and sealed with the DAPI-containing anti-fluorescence-quenching agent (P0131, Beyotime, Shanghai, China). Imaging was performed using the Leica confocal microscope (STELLARIS, Leica, Hessen, Germany) and analyzed using Leica LAS X software.

### 2.9. Statistical Analysis

The results are expressed as the mean ± SEM. GraphPad Prism version 8.0.2 was used for statistical analysis and graphs. One-way ANOVA was used to compare multiple groups. Tukey’s post hoc test was performed only if F reached *p* < 0.05 with homogeneity of variance. *p*-values < 0.05 were considered statistically significant.

## 3. Results

### 3.1. Blood Glucose Levels and Corresponding Symptoms after Insulin Overdose

Following the insulin overdose injection, blood glucose levels decreased significantly over time, and various symptoms appeared, as shown in Figure 1B and Table 1. When blood glucose dropped below 1.5 mmol/L (27 mg/dL), severe symptoms were observed, including irregular convulsions, hypermyotonia, eclampsia, opisthotonos, etc. The blood glucose levels in the insulin overdose group decreased further until death. By contrast, after the injection of glucose, the blood glucose levels in the recovery group increased rapidly to more than 3.9 mmol/L (70 mg/dL); the symptoms gradually disappeared, and all the rats survived.

### 3.2. Effects of Insulin Overdose on Biochemical Indexes

**Figure 2:**
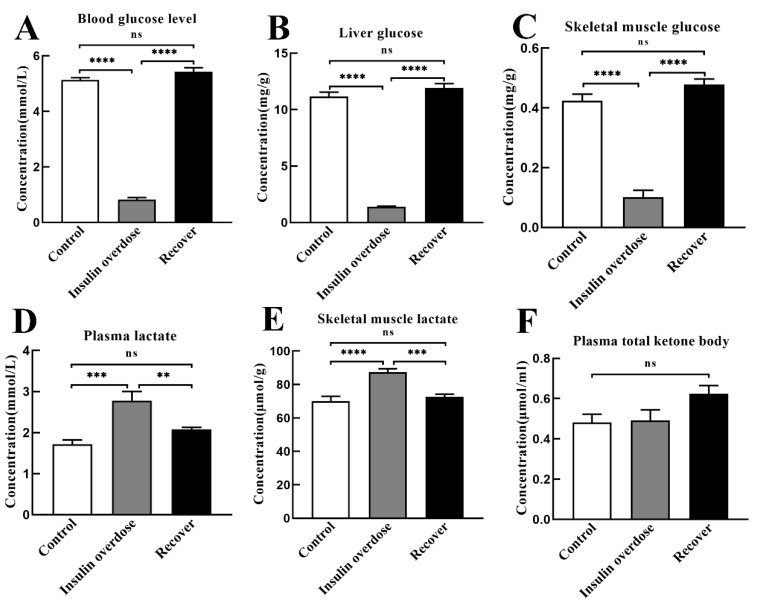
Energy index determination. (**A**–**C**) Glucose levels in plasma, liver, and skeletal muscle of each group; (**D**,**E**) Lactate levels in plasma and skeletal muscle of each group; (**F**) Plasma ketone body levels of each group. Data are expressed as mean ± SEM, ns *p* > 0.05, ** *p* < 0.01, *** *p* < 0.001, **** *p* < 0.0001, One-way ANOVA.

As shown in Figure 2A–C, compared with the control group, glucose levels in plasma, liver, and muscle tissue were significantly decreased in the insulin overdose group (*p* < 0.0001). After 3 days of recovering from an insulin overdose, glucose levels in the blood, liver, and muscle tissue returned to normal. As shown in Figure 2D,E, lactate levels in the skeletal muscle and plasma were significantly increased in the insulin overdose group compared with the control group (muscle *p* < 0.0001, plasma *p* < 0.001). In the recovery group, lactate levels in skeletal muscle and plasma returned to normal. The glucose consumption and lactate accumulation indicated that glycolysis increased after the insulin overdose. In addition, the plasma total ketone body level in the recovery group was slightly increased, but there was no significant difference compared with the other groups (*p* > 0.05), Figure 2F.

**Figure 3:**
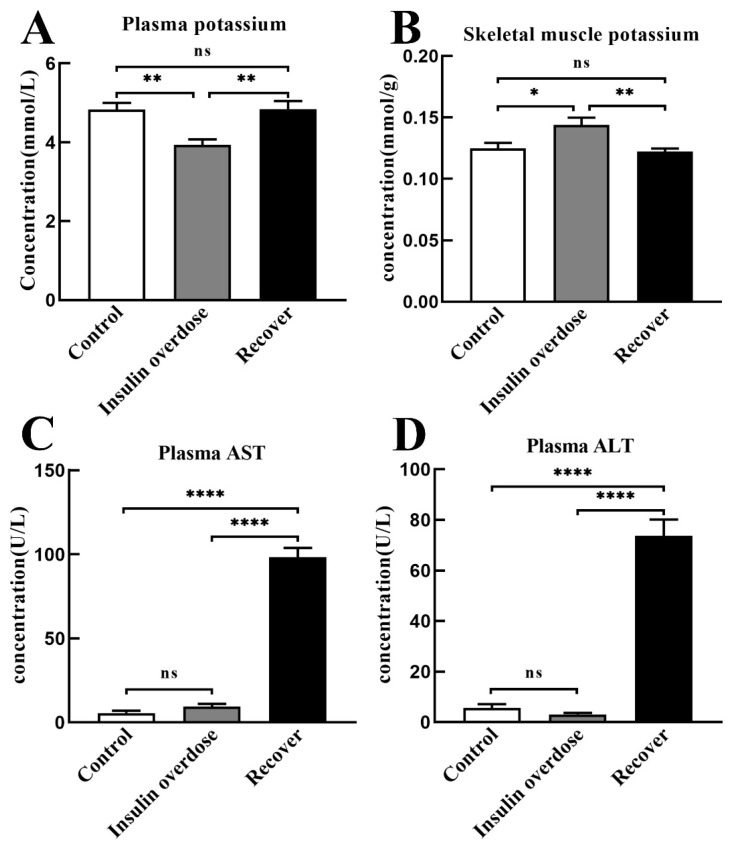
Other biochemical indexes. (**A**,**B**) Potassium levels in plasma and skeletal muscle of each group; (**C**,**D**) Plasma AST and ALT levels in each group. AST, Aspartate aminotransferase; ALT, alanine aminotransferase. Data are expressed as mean ± SEM, ns *p* > 0.05, * *p* < 0.05, ** *p* < 0.01, **** *p* < 0.0001, One-way ANOVA.

As shown in Figure 3A,B, compared with the other groups, the plasma potassium level decreased in the insulin overdose group (*p* < 0.01), while the potassium level in skeletal muscle was increased in the insulin overdose group (*p* < 0.05). These data indicate that insulin overdose alters potassium distribution in plasma and the skeletal muscle.

As shown in Figure 3C,D, compared with the other groups, plasma alanine aminotransferase (ALT) and aspartate transaminase (AST) in the recovery group were significantly increased (*p* < 0.0001), indicating that liver function was damaged after insulin overdose and glucose resuscitation.

### 3.3. Effects of Insulin Overdose on Glucose Metabolic Hormones

**Figure 4:**
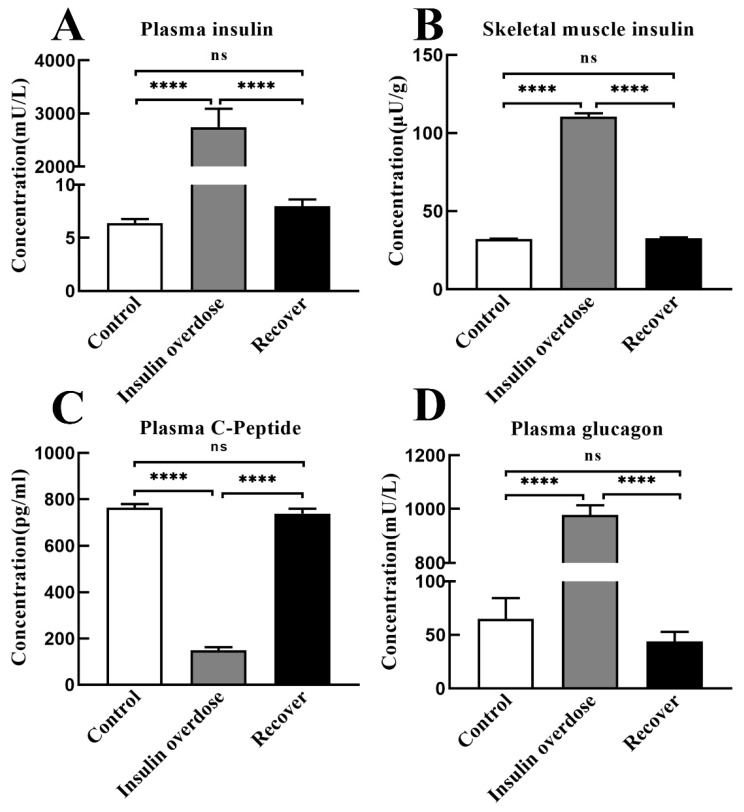
Hormone Levels. (**A**,**B**) Insulin levels in plasma and skeletal muscle of each group; (**C**) Plasma C-peptide levels in each group; (**D**) Plasma glucagon levels in each group. Data are expressed as mean ± SEM, ns *p* > 0.05, **** *p* < 0.0001, One-way ANOVA.

As shown in Figure 4A,B, compared with the other groups, the insulin levels of plasma and skeletal muscle in the insulin overdose group significantly increased (*p* < 0.0001). After recovery, the insulin levels in plasma and skeletal muscle decreased to the control level (*p* > 0.05). Plasma C-peptide levels in the insulin overdose group decreased significantly compared with the control group (*p* < 0.0001); after recovery, plasma C-peptide levels to returned to the control group (*p* > 0.05), as shown in Figure 4C. In addition, plasma glucagon levels in the insulin overdose group were significantly increased compared with the control group (*p* < 0.0001), but plasma glucagon levels in the recovery group decreased to the control level (*p* > 0.05), as shown in Figure 4D. These hormone levels indicate that after insulin overdose, endogenous insulin secretion is suppressed, while glucagon secretion is stimulated sharply.

### 3.4. Glycogen Deposition after Insulin Overdose and Glucose Recovery

**Figure 5:**
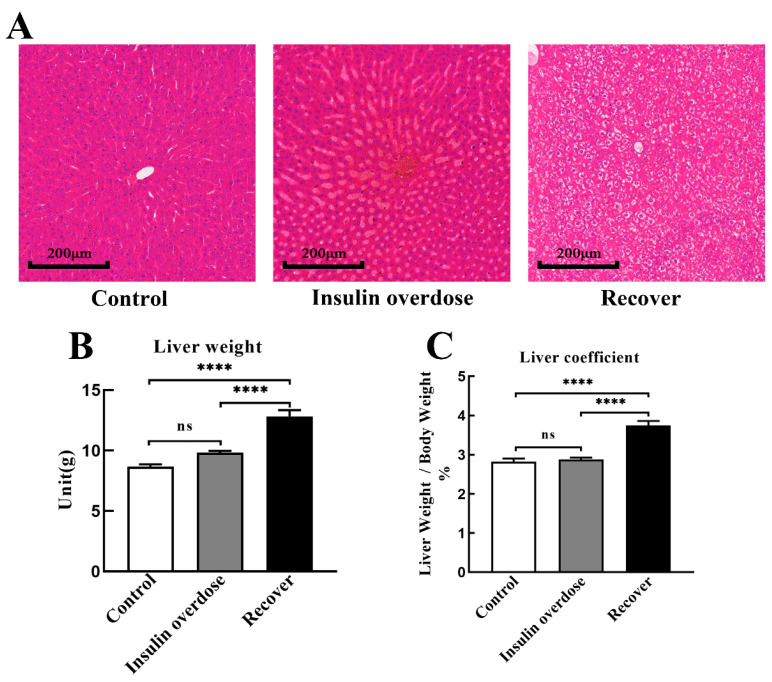
Liver weight and HE staining. (**A**) Representative images of liver HE staining in each group, scale 200 μm; (**B**,**C**) Liver weight and liver coefficient in each group. Data are expressed as mean ± SEM, ns *p* > 0.05, **** *p* < 0.0001, One-way ANOVA.

**Figure 6:**
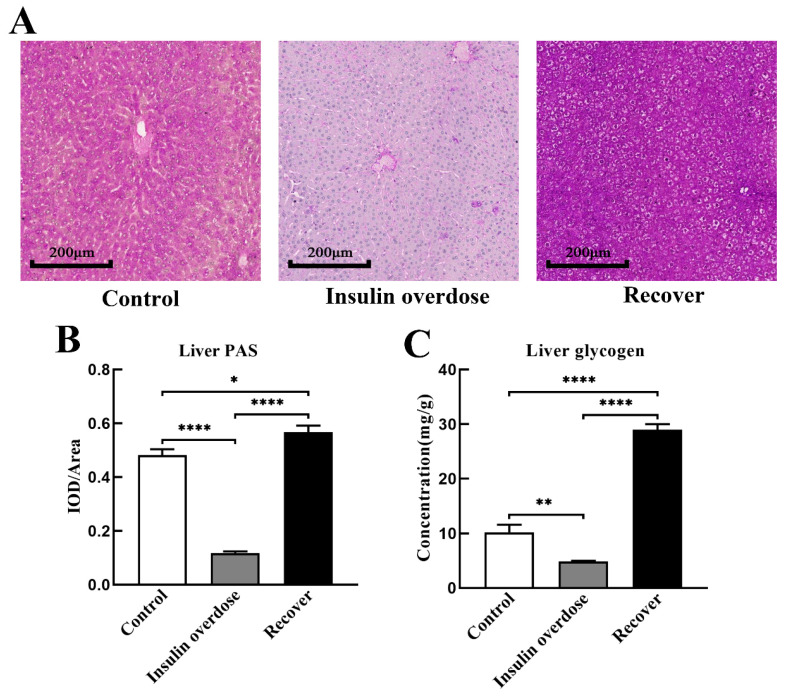
Liver PAS staining and glycogen quantification. (**A**) Representative images of liver PAS staining in each group, scale 200 μm; (**B**) The ratio of IOD and the distribution area of liver PAS staining were calculated using Image J software in each group; (**C**) Liver glycogen was quantitatively detected in each group. Data are expressed as mean ± SEM, * *p* < 0.05, ** *p* < 0.01, **** *p* < 0.0001, One-way ANOVA.

**Figure 7:**
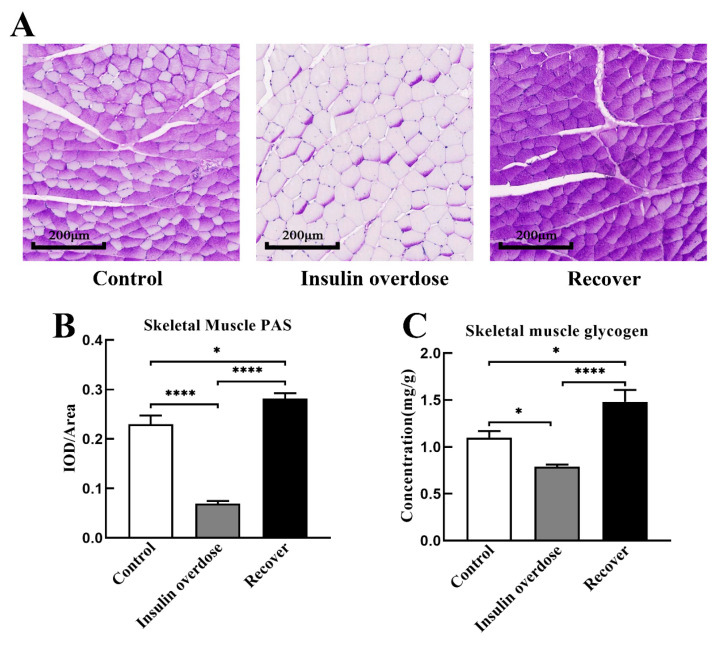
Skeletal muscle PAS staining and glycogen quantification. (**A**) Representative images of skeletal muscle PAS staining in each group, scale 200 μm; (**B**) The ratio of IOD and the distribution area of skeletal muscle PAS staining was calculated using Image J software in each group; (**C**) Skeletal muscle glycogen was quantitatively detected in each group. Data are expressed as mean ± SEM, * *p* < 0.05, **** *p* < 0.0001, One-way ANOVA.

Liver H&E staining, as shown in Figure 5A, resulted in the congestion of the central hepatic vein and hepatic sinuses in the insulin overdose group, including swelling and balloon-like changes in hepatocytes in the recovery group. As shown in Figure 5B,C, there was no significant change in the liver mass and liver coefficient in the insulin overdose group compared with the control group (*p* > 0.05), while the liver mass and liver coefficient were significantly increased in the recovery group (*p* < 0.0001). Compared with the control group, as shown in Figure 6A–C, liver glycogen in the insulin overdose group decreased significantly (PAS staining *p* < 0.0001, glycogen quantification *p* < 0.01), while liver glycogen in the recovery group increased significantly (PAS staining *p* < 0.05, glycogen quantification *p* < 0.0001). Compared with the control group, as shown in Figure 7A–C, skeletal muscle glycogen in the insulin overdose group decreased significantly (PAS staining *p* < 0.0001, glycogen quantification *p* < 0.05), while skeletal muscle glycogen in the recovery group increased significantly (PAS staining *p* < 0.05, glycogen quantification *p* < 0.05). These data indicate that glycogen in both the liver and skeletal muscle decreased after an insulin overdose. However, the glycogen of the liver and skeletal muscle increased after insulin overdose and glucose resuscitation, and hepatocyte degeneration and balloon-like changes, resulting in glycogenic hepatopathy.

### 3.5. Insulin Overdose Activates the PI3K-AKT Signaling Pathway in Skeletal Muscle

**Figure 8:**
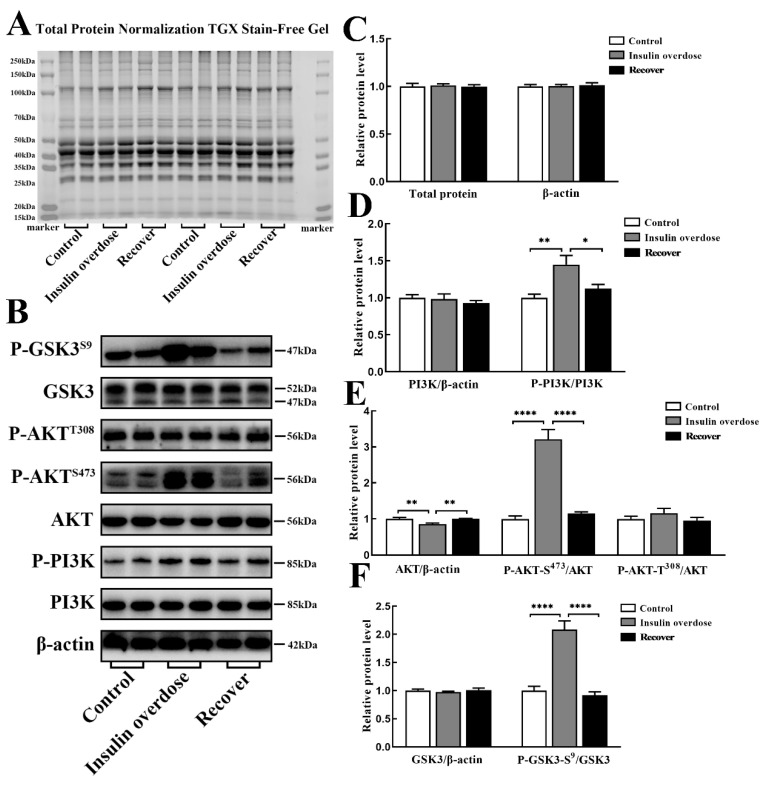
Protein levels of insulin signaling pathway in skeletal muscle. (**A**) Total protein normalization and β-actin internal reference in each group; (**B**) Band images of key proteins in each group; (**C**–**F**) Relative levels of key proteins in each group. Data are expressed as mean ± SEM, * *p* < 0.05, ** *p* < 0.01, **** *p* < 0.0001, One-way ANOVA.

AKT is a key protein that regulates cellular metabolism. As shown in Figure 8A–C, there were no significant changes in the total protein loading amount and the expression of the reference β-actin in each group. As shown in Figure 8B,D, compared with the other groups, the ratio of P-PI3K to total PI3K in the insulin overdose group was significantly increased (*p* < 0.01 VS control group, *p* < 0.05 VS recover group). As shown in Figure 8B,E, compared with the other groups, the ratio of P-AKT-Ser473 to total AKT was significantly increased in the insulin overdose group (*p* < 0.0001 VS control group, *p* < 0.0001 VS recover group), while the ratio of P-AKT-Thr308 to the total AKT was not significantly changed in the hypoglycemia group (*p* > 0.05). As shown in Figure 8B,F, compared with the other groups, the ratio of P-GSK3-Ser9 to total GSK3 was significantly increased in the insulin overdose group (*p* < 0.0001 VS control group, *p* < 0.0001 VS recover group). These data indicate that insulin overdose significantly activates the PI3K-AKT signaling pathway in skeletal muscle and stimulates the ability of glycogen synthesis.

### 3.6. Insulin Overdose Promotes GLUT4 and Na^+^-K^+^-ATPase Translocation in Skeletal Muscle

**Figure 9:**
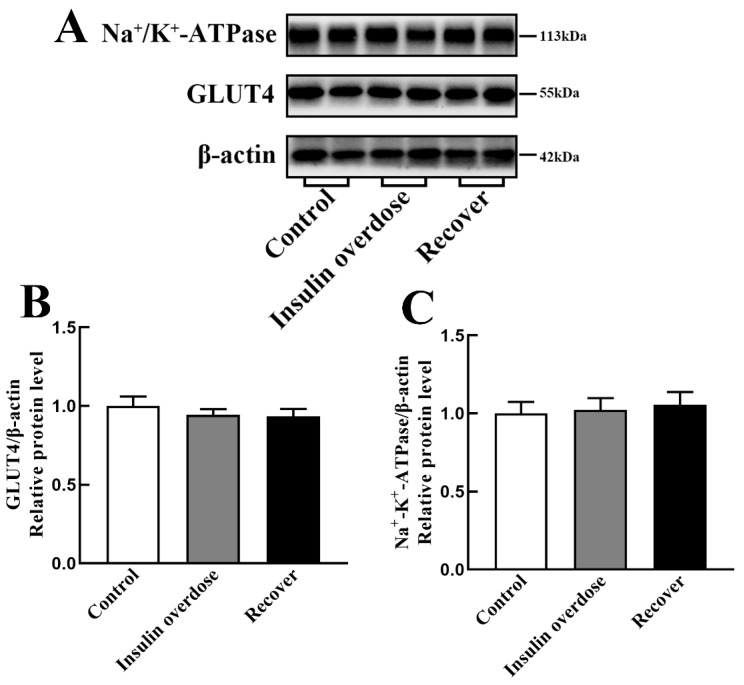
GLUT4 and Na^+^/K^+^-ATPase in skeletal muscle. (**A**) Band images of GLUT4 and Na^+^/K^+^-ATPase in each group; (**B**,**C**) Relative levels of GLUT4 and Na^+^/K^+^-ATPase in each group. Data are expressed as mean ± SEM, One-way ANOVA.

**Figure 10:**
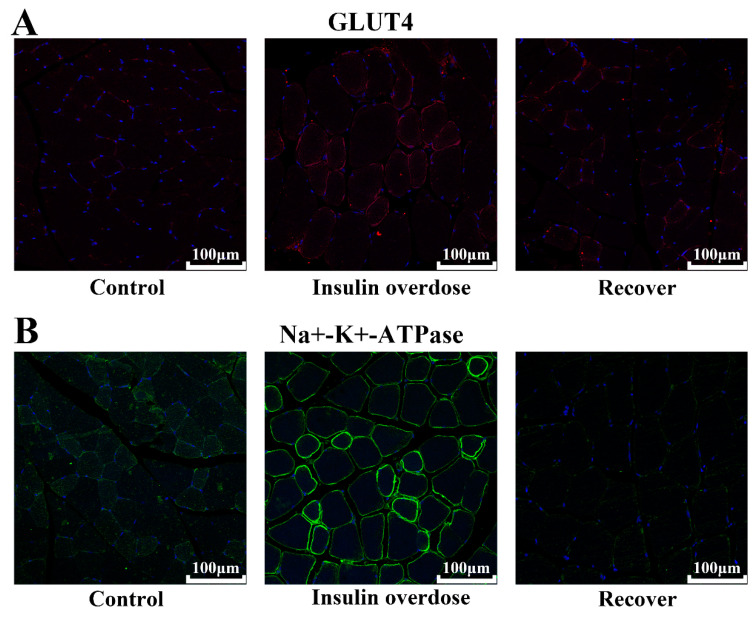
Immunofluorescence images in each group. (**A**) Immunofluorescence images of GLUT4 in skeletal muscle, scale 100 μm; (**B**) Immunofluorescence images of Na^+^/K^+^-ATPase in skeletal muscle, scale 100 μm.

**Figure 11:**
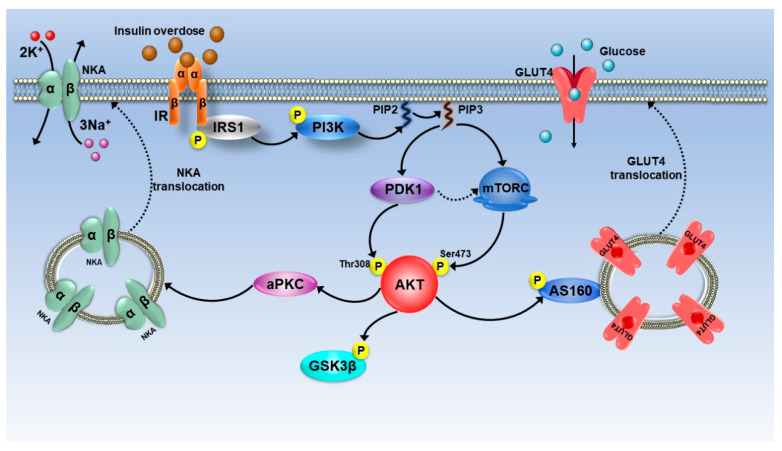
Schematic diagram of the regulation of GLUT4 translocation, Na^+^/K^+^-ATPase translocation, and glycogen synthase kinase phosphorylation after insulin overdose in skeletal muscle. IR: insulin receptor; IRS1: insulin receptor substrate 1; PI3K: Phosphoinositide-3-Kinase; PIP2: phosphatidylinositol-4,5-bisphosphate; PIP3: phosphatidylinositol-3,4,5-bisphosphate; PDK1: Phosphoinositide-dependent kinase; mTORC: Mammalian Target of Rapamycin; AKT: Protein kinase B; aPKC: atypical Protein kinase C; AS160: TBC1D4, TBC1 domain family, member 4; GSK3β: glycogen synthase kinase 3 beta; NKA: Na^+^/K^+^ ATPase 1; GLUT4: solute carrier family 2 (facilitated glucose transporter), member 4.

WB results, as shown in Figure 9A–C, with GLUT4 and Na^+^-K^+^-ATPase expression levels were not significantly different among all groups (*p* > 0.05). However, compared with the other groups, confocal microscopy immunofluorescence images, as shown in Figure 10, indicated that the signals of GLUT4 and Na^+^-K^+^-ATPase on the sarcolemma were significantly increased in the insulin overdose group. The above data and the phosphorylation of PI3K-AKT suggest that insulin overdose stimulates GLUT4 and Na^+^-K^+^-ATPase translocation to the sarcolemma through the PI3K-AKT signaling pathway. Figure 11 shows the schematic diagram of the membrane translocation of GLUT4 and Na^+^-K^+^-ATPase stimulated by insulin overdose, which was drawn by ourselves.

## 4. Discussion

Insulin has a variety of effects on peripheral tissues, such as promoting glucose transport from extracellular to intracellular, regulating cellular glucose metabolism, promoting substance synthesis, inhibiting glycogenolysis, inhibiting gluconeogenesis, etc. [24,25]. The physiological effects of insulin are mainly realized through the PI3K-AKT signaling pathway, in which AKT plays an important role in substance metabolism [24,26,27]. As shown in Figure 11, insulin binds to the insulin receptor (IR), phosphorylates the tyrosine site of insulin receptor substrate 1 (IRS1) in the skeletal muscle, and then activates Phosphoinositide-3-Kinase (PI3K). Activated PI3K catalyzes Phosphatidylinositol-4,5-bisphosphate (PIP2) to Phosphatidylinositol-3,4,5-bisphosphate (PIP3), which acts as a second messenger to activate Protein kinase B (AKT) through phosphoinositide-dependent kinase (PDK1) and the Mammalian Target of Rapamycin (mTORC), respectively [26,27,28]. Phosphorylation at Thr308 is required for AKT activation, while phosphorylation at Ser473 is required for complete AKT activation [27,29]. In this study, as shown in Figure 8, we found that the phosphorylation levels of PI3K and Akt were significantly increased in skeletal muscle cells after insulin overdose, which verified that insulin overdose also exerts its effects mainly through the PI3K-Akt signaling pathway in skeletal muscle cells.

Glucose transporter 4, as an insulin-sensitive glucose transporter, is mainly distributed in the skeletal muscle and adipose tissue [30,31]. Without insulin stimulation, more than 95% glut4 is stored in cells as vesicle GSV [30,32,33]. When insulin stimulates the cells, the downstream proteins are activated through the PI3K-Akt signaling pathway [33,34]. This leads to GLUT4 vesicles losing their anchoring function and translocating to the cell membrane in the form of vesicles, thereby promoting glucose absorption and playing a vital role in lowering blood glucose levels [30,33,35]. The half-life of insulin in the body is only 4–6 min [2,25]. After insulin binds to the receptor, the signaling pathway is activated, and the insulin–receptor complex is rapidly degraded after internalization [2,32]. Therefore, physiological doses of insulin do not cause the sustained translocation of GLUT4 to the membrane of skeletal muscle cells [32,33]. In this study, as shown in Figure 9B, we found no significant difference in the expression of GLUT4 between the insulin overdose group and the control group. However, as shown in Figure 8B and Figure 10A, the intensified immunofluorescence signal of GLUT4 on the skeletal sarcolemma and the activation of the PI3K-Akt signaling pathway following insulin overdose suggested that excessive insulin facilitated the translocation of GLUT4 to the muscle membrane, leading to increased glucose absorption in the skeletal muscle. Furthermore, excess insulin can maintain the continuous translocation of GLUT4 to skeletal sarcolemma, and considering the specific effect of insulin on GLUT4, the detection of GLUT4 signal intensity in skeletal sarcolemma could be useful for the forensic diagnosis of insulin overdose.

Skeletal muscle stores more than 70% of the total body K^+^, with a total amount of about 2600 mmol, which is 46 times the total extracellular K^+^ and 236 times the total plasma K^+^, and contains abundant Na^+^-K^+^-ATPase [36,37]. Therefore, slight K^+^ changes in skeletal muscle can cause significant changes in plasma K^+^ [36,38]. K^+^ homeostasis on a moment-to-moment basis is mainly achieved by the transmembrane transport of extracellular K^+^ to skeletal muscle [38,39,40]. Studies have shown that insulin may activate the PI3K-Akt signaling pathway and promote the translocation of intracellular vesicles containing Na^+^-K^+^-ATPase to the skeletal sarcolemma through atypical protein kinase C (aPKC) [40,41]. Interestingly, it has also been shown that muscle contractions during intense exercise or during epilepsy promote the diffusion of K+ from the muscles to the capillaries, thereby raising blood potassium levels [36,41]. In this study, as shown in Figure 9C, we found no significant difference in the expression level of Na^+^-K^+^-ATPase between the insulin overdose group and the control group. However, as shown in Figure 10B, there was an enhanced immunofluorescence signal of Na^+^-K^+^-ATPase on the skeletal sarcolemma, suggesting that excessive insulin activates the PI3K-Akt signaling pathway, leading to the translocation of Na^+^-K^+^-ATPase to the skeletal sarcolemma and subsequently increasing the transport of blood potassium into the muscles. It is important to emphasize that the decrease in blood potassium levels after insulin overdose is primarily attributed to the skeletal muscle-mediated translocation of potassium rather than absolute potassium loss. Clinical cases have provided evidence that aggressively supplementing potassium in response to initial hypokalemia during the early stages of an insulin overdose can lead to hyperkalemia once the underlying cause of the insulin overdose is resolved [42,43]. Therefore, it is necessary to prolong the monitoring of the plasma potassium level and consider conservative potassium supplementation in clinical practice for the decrease in plasma potassium induced by insulin overdose. In addition, due to the relatively specific effect of insulin on Na^+^-K^+^-ATPase, it is worthwhile to further investigate the detection of the signal intensity of Na^+^-K^+^-ATPase in skeletal sarcolemma for the forensic diagnosis of insulin overdose.

Studies have shown that the body activates counterregulatory systems to prevent hypoglycemia in response to decreased blood glucose levels [44,45,46]. After an exogenous insulin overdose, the secretion of endogenous insulin decreases, and the release of glucose-raising hormones increases, among which glucagon plays a major role [44,45,47]. Furthermore, hypoglycemia can increase the ability of glucagon to produce glucose in the liver [48,49], and the dual effect of hypoglycemia and glucagon surpasses the inhibitory impact of insulin, resulting in enhanced glycogen decomposition but has no significant effect on gluconeogenesis [45,48,50]. In this study, as shown in Figure 4A,D, plasma insulin levels and glucagon levels were significantly increased after insulin overdose, while liver glycogen and glucose contents were significantly decreased, confirming the hepatic glycogen depletion caused by insulin overdose. Previous studies have shown that, during intense exercise, glucose is the only fuel for muscles, while muscles produce large amounts of lactate that diffuse along the concentration gradients [51,52,53,54]. In this study, symptoms such as persistent muscle twitching during insulin overdose may lead to substantial glucose consumption and the production of significant amounts of lactic acid in the skeletal muscle. Although insulin overdose promotes GLUT4 cell membrane translocation and glycogen synthase kinase activation in the skeletal muscle, it eventually leads to glycogen consumption in the skeletal muscle under the dual effects of energy consumption and the negative feedback regulation mechanism after insulin overdose. Therefore, the detection of glycogen levels in the liver and skeletal muscle is beneficial for the forensic diagnosis of insulin overdose, but it needs to be differentiated from other causes of death.

When endogenous insulin is released from the pancreas, equimolar amounts of the C-peptide are released simultaneously, and the C-peptide is metabolized and cleared more slowly than insulin, so the C-peptide can be used to assess the level of endogenous insulin secretion [55,56,57]. In the normal body, the ratio of insulin to C-peptide (I:C) is less than 1 [55]. When hyperinsulinemia occurs, such as pancreatic β-cell tumors and the rare insulin autoimmune syndrome, the insulin level and C-peptide level increase simultaneously, and the ratio of insulin to C-peptide (I:C) is still less than one [55,58]. However, when exogenous insulin is injected into the body, endogenous insulin secretion is inhibited, resulting in a decrease in the C-peptide level, and the ratio of the insulin level to C-peptide (I:C) is greater than one [55,57]. In this study, as shown in Figure 4C, the level of C-peptide decreased significantly after insulin overdose, and the ratio of insulin to the C-peptide (I:C) was much greater than one, which could be diagnosed as an exogenous insulin overdose. Therefore, both forensically and clinically, measurements of insulin and the C-peptide, as well as the ratio of insulin to the C-peptide (I:C), can be used to determine whether an insulin overdose is endogenous or exogenous.

For the immunological detection of insulin, it is necessary to consider the influence of insulin analog cross-reactions [59,60]. In this study, the Mercodia Iso-Insulin Elisa kit validated by an insulin analog cross-reaction was used for detection to exclude the interference of a cross-reaction on the detection value. Studies have shown that insulin is easily distributed widely throughout the body through blood vessels [61,62]. Clinically, hypoglycemia caused by an exogenous insulin overdose can be diagnosed via the simultaneous detection of an absolute increase in insulin, a decrease in the C-peptide level, and a decrease in the blood glucose level of <2.8 mmol/L (50 mg/dL) [63]. However, in forensic practice, insulin in postmortem blood is easily degraded by hemolysis, corruption, and other influences, which makes it extremely difficult to detect insulin in postmortem blood [64]. In addition, postmortem blood glucose shows a wide range of fluctuations, resulting in blood glucose parameters that are also of no value in forensic pathology [65]. Interestingly, Duckworth found that the muscle is the third major organ for insulin metabolism [66], and Bryant confirmed that insulin is more stable in acidic media [67]. In this study, as shown in Figure 2E and Figure 4B, both insulin and lactate levels were increased in the skeletal muscle after insulin overdose. Given the ample sample size of the muscle and the acidic environment provided by postmortem glycolysis, there is great potential for forensic insulin detection from muscle.

Glycogenic hepatopathy is a rare complication of poor glycemic control in patients with type 1 diabetes mellitus [68,69], which is pathologically characterized by elevated liver transaminase, hepatomegaly, and glycogen deposition [70,71]. In this study, rats developed glycogenic hepatopathy similar to those in type 1 diabetes after resuscitation and insulin overdose. In the recovery group, as shown in Figure 3C,D, Figure 5 and Figure 6, AST and ALT were significantly increased, the liver weight and liver coefficient were significantly increased, HE staining showed hepatomegaly and ballooning degeneration, and PAS staining and glycogen measurement confirmed that liver glycogen was significantly increased. Studies have shown that transient glycogenic hepatopathy generally does not cause persistent liver damage, but it is easily misdiagnosed by clinicians as non-alcoholic fatty liver disease (NAFLD) in patients with type 2 diabetes [70,72], which requires differential diagnosis by clinicians.

## 5. Conclusions

In conclusion, our study shows that insulin overdose also plays a role in skeletal muscle cells mainly through the PI3K-Akt signaling pathway, and the detection of GLUT4 and Na^+^-K^+^-ATPase signals in the skeletal muscle cell membrane is helpful for the forensic diagnosis of insulin overdose. Glycogen detection in the liver and skeletal muscle is helpful for the forensic diagnosis of insulin overdose, but it needs to be differentiated from other causes of death. Skeletal muscle has great potential as a substitute for blood to detect an insulin overdose, and the ratio of insulin to C-peptide (I:C) can be used to determine whether it is an exogenous insulin overdose.

## Figures and Tables

**Table 1:** Blood glucose levels and corresponding symptoms in rats. Data are expressed as the mean ± SEM.

Time	Blood Glucose		Symptoms
	$\mathrm{mmol}/L\;\bar{X}\pm$ SEM	$\mathrm{mg}/\mathrm{dL}\;\bar{X}\pm \mathrm{SEM}$	
0 h	5.5 ± 0.4	99.0 ± 7.2	Normal behavior
0.5 h	2.5 ± 0.4	45.0 ± 7.2	Blunted response; hypodynamia; hypotonia
1 h	1.5 ± 0.3	27.0 ± 5.4	Irregular convulsions; hypermyotonia
1.5 h	1.2 ± 0.2	21.6 ± 3.6	Eclampsia; opisthotonos
2 h	0.9 ± 0.2	16.2 ± 3.6	Collapse; uroclepsia; near-death
2.5 h	0.8 ± 0.1	14.4 ± 1.8	Death

## Data Availability

Data are available upon request to the corresponding author.
